# Anti-prostate cancer protection and therapy in the framework of predictive, preventive and personalised medicine — comprehensive effects of phytochemicals in primary, secondary and tertiary care

**DOI:** 10.1007/s13167-022-00288-z

**Published:** 2022-07-08

**Authors:** Alena Mazurakova, Marek Samec, Lenka Koklesova, Kamil Biringer, Erik Kudela, Raghad Khalid Al-Ishaq, Martin Pec, Frank A. Giordano, Dietrich Büsselberg, Peter Kubatka, Olga Golubnitschaja

**Affiliations:** 1 Clinic of Obstetrics and Gynecology, Jessenius Faculty of Medicine, Comenius University in Bratislava, 036 01 Martin, Slovakia; 2 Department of Pathological Physiology, Jessenius Faculty of Medicine, Comenius University in Bratislava, 036 01 Martin, Slovakia; 3 Department of Physiology and Biophysics, Weill Cornell Medicine-Qatar, Qatar Foundation, Education City, Doha, Qatar; 4 Department of Medical Biology, Jessenius Faculty of Medicine, Comenius University in Bratislava, 036 01 Martin, Slovakia; 5 Department of Radiation Oncology, University Hospital Bonn, Rheinische Friedrich-Wilhelms-Universität Bonn, Bonn, Germany; 6 Predictive, Preventive and Personalised (3P) Medicine, Department of Radiation Oncology, University Hospital Bonn, Rheinische Friedrich-Wilhelms-Universität Bonn, 53127 Bonn, Germany

**Keywords:** Prostate cancer management, Metastatic disease, Predictive Preventive Personalised Medicine (PPPM/3PM), Sub-optimal health condition, Health-to-disease transition, Risk assessment, Phenotyping, Primary secondary tertiary care, Phytochemicals, Plant-based food, Clinical trials, Molecular mechanisms, ROS, Stress, Mitochondrial health, Anti-cancer protection, Radiation and chemotherapy, Tailored treatments, Cost-efficacy, COVID-19, Silibinin, Health policy

## Abstract

According to the GLOBOCAN 2020, prostate cancer (PCa) is the most often diagnosed male cancer in 112 countries and the leading cancer-related death in 48 countries. Moreover, PCa incidence permanently increases in adolescents and young adults. Also, the rates of metastasising PCa continuously grow up in young populations. Corresponding socio-economic burden is enormous: PCa treatment costs increase more rapidly than for any other cancer. In order to reverse current trends in exploding PCa cases and treatment costs, pragmatic decisions should be made, in favour of advanced populational screening programmes and effective anti-PCa protection at the level of the health-to-disease transition (sub-optimal health conditions) demonstrating the highest cost-efficacy of treatments. For doing this, the paradigm change from reactive treatments of the clinically manifested PCa to the predictive approach and personalised prevention is essential.

Phytochemicals are associated with potent anti-cancer activity targeting each stage of carcinogenesis including cell apoptosis and proliferation, cancer invasiveness and metastatic disease. For example, their positive effects are demonstrated for stabilising and restoring mitochondrial health quality, which if compromised is strongly associated with sub-optimal health conditions and strong predisposition to aggressive PCa sub-types. Further, phytochemicals significantly enhance response of cancer cells to anti-cancer therapies including radio- and chemotherapy. Evident plant-based mitigation of negative side-effects frequently observed for conventional anti-cancer therapies has been reported. Finally, dual anti-cancer and anti-viral effects of phytochemicals such as these of silibinin have been demonstrated as being highly relevant for improved PCa management at the level of secondary and tertiary care, for example, under pandemic conditions, since PCa-affected individuals per evidence are highly vulnerable towards COVID-19 infection.

Here, we present a comprehensive data analysis towards clinically relevant anti-cancer effects of phytochemicals to be considered for personalised anti-PCa protection in primary care as well as for an advanced disease management at the level of secondary and tertiary care in the framework of predictive, preventive and personalised medicine.

## Preamble

Prostate cancer (PCa) represents one of the most frequent cancer types in men in both incidence and mortality [1]. Following lung cancer, PCa is the second most frequently occurring cancer in men globally. According to the GLOBOCAN statistics presented for 2020, 1,414,259 new PCa cases accounted for 14.1% of all cancer sites in men. Moreover, PCa was the most often diagnosed cancer in men in 112 countries. Similarly, in 2020 PCa accounted for 375,304 new deaths and thus represented the fifth most frequent cause of cancer death in men accounting for 6.8%. PCa was the leading cause of cancer-related death in 48 countries. The highest PCa incidence rates are found in Northern and Western Europe, The Caribbean, Australia/New Zealand, Northern America, and Southern Africa while the lowest incidence rates are in Asia and Northern Africa. Further, the highest mortality rates are in the Caribbean, Central and South America (e.g. Ecuador, Venezuela, Chile), and Sweden. The role of Western African ancestry in the modulation of PCa risk is supported by the highest global incidence of PCa in black men in the USA and Caribbean. To this end, national PCa diagnostics standards strongly contribute to PCa incidence-to-mortality statistics varying between countries and continents [2]. Moreover, PCa incidence is permanently increasing in adolescents and young adults (aged 15–40 years). Also, the rates of metastasising PCa are steadily growing up in the young population [3, 4].

Socio-economic burden is enormous: PCa treatment costs increase more rapidly than for any other cancer [3]. To this end, anti-cancer mRNA-based therapy is a promising approach based on experience collected during the last couple of months from the anti-COVID-19 vaccination. However, consideration of short- and long-term effectiveness of this kind of vaccination and its potential side effects will take years or even decades, in order to optimize the treatment condition for each cancer subtype individually [3]. In order to reverse current trends in exploding PCa statistics and treatment costs, pragmatic decisions should be made, to advance populational screening programmes and to force an effective anti-Pca protection at the level of the health-to-disease transition (sub-optimal health conditions) demonstrating the highest cost-efficacy of treatments. For doing this, the paradigm change from reactive treatments of the clinically manifested PCa to a predictive approach and personalised prevention [3].

Here, we present a comprehensive data analysis towards clinically relevant anti-cancer effects of phytochemicals [5–9] to be considered for personalised anti-PCa protection in primary care as well as for advanced disease management at the level of secondary and tertiary care in the framework of predictive, preventive and personalised medicine (PPPM/3PM).

## Plant-based anti-cancer intervention — the general view

Phytochemicals are secondary plant metabolites, non-nutritive compounds produced by plants [10]. Main phytochemical classes are including polyphenols, carotenoids, alkaloids, and organosulfur compounds as recently analysed by Mazurakova et al. (2022) [9]. Current evidence highlights potent anti-cancer effects of phytochemicals and plant-based anti-cancer intervention [5, 7, 11, 12]. Recent reviews and original articles discuss the anti-cancer effects of phytosubstances demonstrated in preclinical in vitro and in vivo evaluations, including remarkable impacts on PCa prevention, inhibition of the disease progression and stimulating effects of phyto-substances on anti-cancer therapies [13–16]. Identification of health beneficiary effects as well as precise mechanism of the anti-cancer action by phytochemicals are essential for associated drug development and recommendations for personalised dietary supplements [5, 9, 10].

Phytochemicals are associated with potent anti-cancer activity affecting each of the multistage process of carcinogenesis, including apoptosis, proliferation, and invasion of cancer cells and related processes of cancer angiogenesis and metastasis [5–7, 17]. Moreover, oxidative stress overload associated with PCa development and progression results from molecular and sub-cellular changes synergistically leading to compromised mitochondrial health quality [18]. The key role of mitochondrial health quality control in the targeted anti-PCa protection can be illustrated by the capacity of apigenin to induce apoptosis of PCa cells in vitro [19]. Noteworthy, prostate tissue analysed in African American men has been associated with reduced mitochondrial DNA (mtDNA) content compared to Caucasian American while men. This may help to explain higher incidence rates and more aggressive PCa subtypes in African American men. Compromised mitochondrial health related to mtDNA depletion results in defective OXPHOS, uncontrolled ROS production, and extensive mutations to mtDNA. Dysfunctional mitochondria are also associated with increased metastatic potential and stemness of PCa cells as well as significant radio-resistance of related prostate malignancies [20].

Abundant evidence indicates a potent role of phytochemicals in both — mitochondrial health quality support on the one hand and on the other hand significantly enhanced response of cancer cells towards anti-cancer therapies including radiotherapy and chemotherapy; also an evident mitigation of negative side-effects frequently observed for conventional anti-cancer therapies have been reported [21–26].

**Fig. 1:**
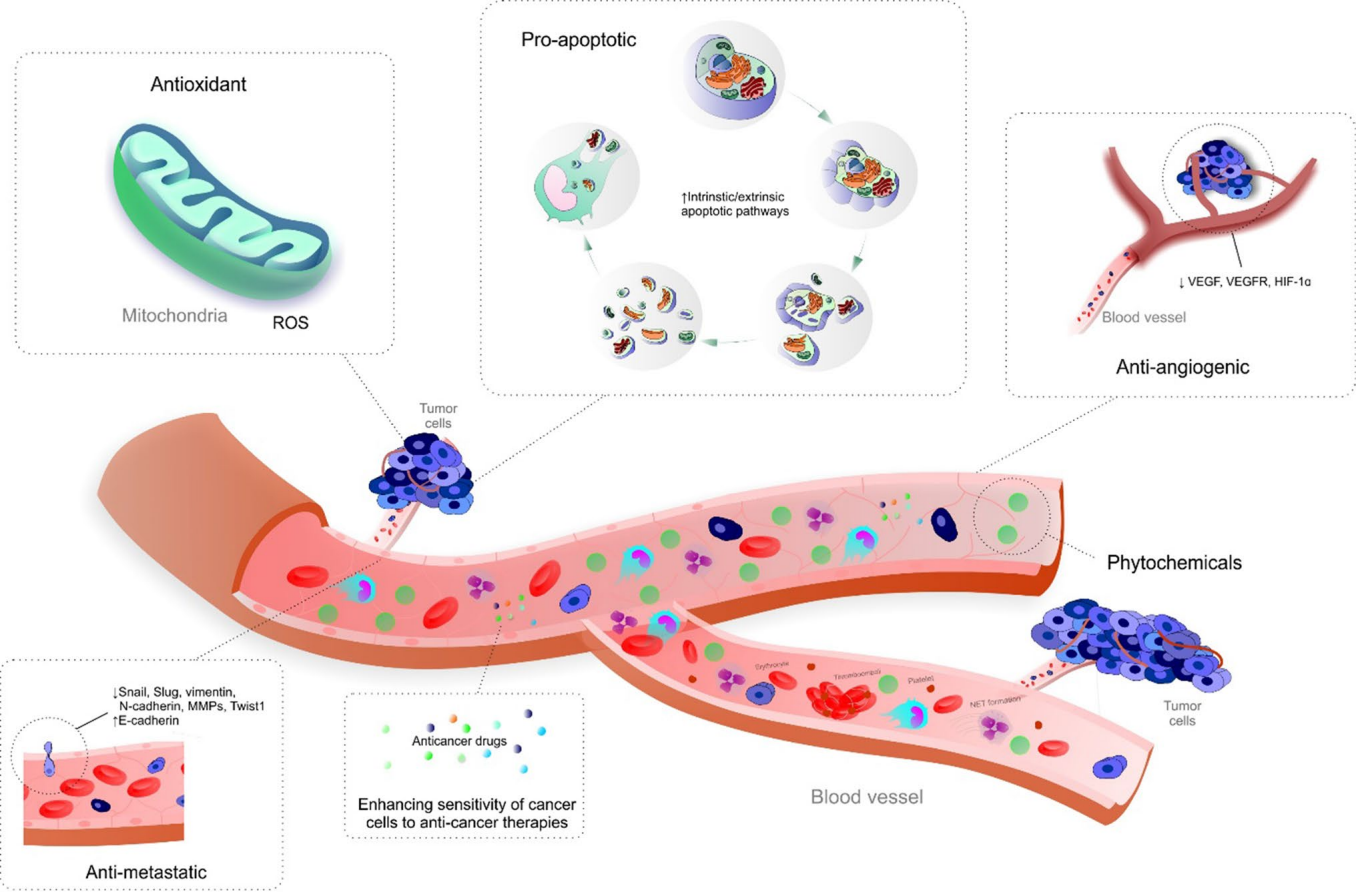
Anti-cancer effects of phytochemicals in PCa: apoptosis, proliferation, invasion, angiogenesis, metastasis, and effect on PCa therapies

Figure 1 depicts the anti-cancer effects of phytochemicals and their impact on each of the processes of carcinogenesis including apoptosis, oxidative stress, angiogenesis, metastasis, and affecting the effectiveness of conventional anti-cancer strategies. As is discussed below, clinical evaluations of anti-cancer capacity of phytochemicals provide evidence of their efficacy in each of the illustrated processes of carcinogenesis in PCa primary and secondary care as well as in combination with conventional anti-cancer therapeutic modalities.

## Protection against clinical manifestation of PCa: phytochemicals for primary care

The preventable nature of most PCa cases represents a platform for effective risk assessment, disease-predisposition, and effective preventive and personalised strategies [1, 3]. For example, pre-cancerous lesions, such as high-grade intraepithelial neoplasia (HGPIN), can be detected years before the progression to PCa. Therefore, targeted prevention and early diagnosis are essential strategies to reduce PCa [27]. Naturally occurring phytochemicals are widely known for their anti-cancer effects on all of the multistep processes of carcinogenesis, including cancer initiation, promotion, and progression [5, 6, 8]. The study published at the end of the twentieth century evaluating the data from three case–control studies points to a potential association between plant foods (green and cruciferous vegetables, tomatoes, and beans) and whole-grain bread and reduced PCa risk [28]. A year later, legumes and specific vegetable categories were suggested to protect against PCa [29]. Moreover, Ambrosini et al. (2008) observed a decreased PCa risk with increasing intake of vegetable rich in vitamin C (bell peppers and broccoli) [30]. Furthermore, as discussed below, current evidence provides the clinical evaluations of anti-cancer effects of other plant-based food subtypes in PCa management.

### Green tea phytochemicals

The main phytochemicals in green tea are known as green tea catechins (GTC), which include epigallocatechin-3-gallate (EGCG), epicatechin (EC), epigallocatechin (EGC), and epicatechin-3-gallate (ECG). Several authors reviewed GTC as effective in reducing PCa risk, especially in Asian populations characterised by increased intake of green tea [31, 32]. However, the overall clinical data neither confirm nor refute the protective effects of green tea against PCa. A case–control study in southeast China that was published in 2004 demonstrated declined risk of PCa with increasing frequency, duration, and quantity of green tea consumption [33]. Similarly, the potential effectiveness of GTC in PCa prevention was supported by McLarty et al. (2009) who demonstrated that the administration of Polyphenon E, a mixture of tea catechins, decreased serum levels of PSA, HGF, and VEGF with no elevation in liver enzymes in men with PCa [34]. Oxidative DNA damage plays an important role in carcinogenesis [35]. Moreover, prostate tissue is suggested to be more vulnerable to oxidative damage due to the fewer DNA repair enzymes, faster cell turnover, and chronic inflammation of prostate epithelial cells. Indeed, 8-hydroxy-2′-deoxyguanosine (8-OHdG) is the product of oxidative damage of the DNA base 2′-deoxyguanosine(dG) [35]. Therefore, 8-OHdG is considered a marker of oxidative stress and has been observed to be expressed more highly in PCa tissue when compared with benign prostate tissues [36]. However, decreased 8-OHdG is associated with human leukocytes in individuals consuming food rich in antioxidants [35]. Indeed, green tea resulted in altered PCa development and progression biomarkers — decreased NFκB in radical prostatectomy tissues, systemic antioxidant effect (reduced urinary 8OHdG), and a small but significant decrease in serum PSA levels [37]. Also, Nguyen et al. (2012) suggested that green tea chemo-preventive abilities in PCa may not be mediated by direct means or occurs without accumulation.

However, the authors concluded the need for long-term interventions, repeated doses for more constant exposure, or evaluations in pre-cancerous models [38]. However, these results must be interpreted with caution due to the inconsistency of the results of other studies. For example, results of the placebo-controlled, randomised clinical trial (2015) evaluating the potential anti-cancer effectiveness of Polyphenon E demonstrated that EGCG accumulated in plasma and was well tolerated but did not reduce PCa risk in men with HGPIN and/or atypical small acinar proliferation (ASAP) [39]. Also, recent 3-week-long pre-prostatectomy intervention (2020) evaluating the combination of quercetin with green tea extract for 4 weeks revealed no significant increase in EGCG or EGC concentrations or decrease in GTP methylation in prostate tissues [40]. Moreover, fatty acid synthase (FAS) catalyses final step in fatty acid synthesis de novo. In tumour cells, the rate of fatty acid synthesis is greater. Also, FAS gene was found to be upregulated by hypoxia in tumour cells. Therefore, overexpressed FAS appears to play important role in PCa [41]. As FAS is hypothesised to be associated with chemo-preventive effects of fish oil and green tea, Zhang et al. (2016) evaluated their effects in PCa patients. However, the results demonstrated no effects of fish oil and green tea supplement (EGCG) administered during a short duration on FAS or Ki67 in PCa [41].

### Carotenoids

The evidence suggests the PCa protective role of tomato or tomato phytochemicals such as lycopene, a non-provitamin A carotenoid [42, 43]. Beynon et al. (2019) demonstrated the efficacy of lycopene in lowering pyruvate levels. Indeed, decreased pyruvate is related to reduced PCa risk as supported by Mendelian randomisation suggesting the association between genetically predicted higher pyruvate levels and increased risk of PCa [44]. Moreover, study results published in 2008 indicated that lycopene may inhibit disease progression in patients with benign prostate hyperplasia [45]. Similarly, *Serenoa repens*, derived from the saw palmetto tree berries, selenium, and lycopene, may exert anti-inflammatory effects that could benefit the treatment of chronic prostatic inflammation in benign prostate hyperplasia and/or PIN/ASAP [46]. The evaluation of the effects of red or yellow tomato paste and purified lycopene resulted in increased circulating lycopene only after consuming red tomato paste and purified lycopene. At the same time, antioxidant status, PSA, and insulin-like growth factor-1 (IGF-1) did not modify by tomato paste consumption. However, upregulated IGFBP-3 and Bax/Bcl-2 ratio and decreased cyclin-D1, p53, and Nrf-2 after ex vivo cell incubation with sera from healthy men who consumed red tomato paste [47]. As recently demonstrated by Fraser et al. (2020), the consumption of canned and cooked tomatoes that contain more available lycopene may reduce the PCa risk. However, the inability to distinguish between PCa molecular subtypes limits the study results [48]. On the contrary, another study demonstrated the role of tomato sauce in the reduction of *TMPRSS2:ERG*-positive PCa [49]. On the contrary, the results of a small pilot, randomised-controlled trial demonstrated no effects of a tomato-enrich diet, lycopene, or green tea to affect serum levels of IGF-I, IGF-II, IGFBP-3, or IGFBP-2 [50]. Paradoxically, Gontero et al. (2015) observed three times higher incidence of PCa at re-biopsy and microRNAs associated with PCa progression in men with primary multifocal HGPIN and/or ASAP administered with high non-toxic doses of lycopene, green tea catechins, and selenium when compared with participants without supplementation. However, the evaluation of three compounds does not allow the precise analysis of individual substances [51]. However, Morgia et al. (2017) did not show the evidence of deleterious effects of selenium and lycopene in increasing PCa risk after 2 years of therapy, nor supported the protective role [52]. Similarly (2011), the associations between serum lycopene and PCa prevention have not been supported in nested case–control study in the Prostate Cancer Prevention Trial either [53]. Several other studies (2007, 2015) reflect no effects of tomato or lycopene on decreasing PCa risk [54, 55].

In addition to lycopene, the evidence on the association between other carotenoids and retinol and PCa risk is inconsistent [56]. Neuhouser et al. (2009) provided modest evidence of the association between increased PCa risk and high-dose β-carotene (30 mg/day) and retinyl palmitate (25,000 IU/day) administered for lung cancer prevention plus at least one other dietary supplement [57]. Moreover, Nash et al. (2015) described an increased PCa risk in men with higher serum retinol and α-carotene [56]. Also, a recent study by Chadid et al. (2022) concluded common circulating carotenoids and retinol as not useful in preventing PCa through the modulation of intraprostatic inflammation [58]. However, serum levels of α-carotene, retinyl esters and lycopene have been demonstrated to be associated with PSA biomarkers in US men and thus could be useful in early PCa detection [59].

### Isoflavones of soy

Soy is a rich source of isoflavones while the main soy isoflavone — genistein — is associated with potent anticancer efficacy [60–62]. It is known that prostate tissue can concentrate genistein and other phytochemicals [63]. In 2012, Lazarevic et al. supported the chemo-preventive role of genistein in PCa demonstrated through the modulation of biomarkers related to prediction and progression of the disease — including reduced KLK4 in tumour cells and a non-significant decrease in androgen and cell cycle–related biomarkers [62]. Importantly, soy isoflavones (administered to PCa patients in a neo-adjuvant setting for 2 weeks before prostatectomy) resulted in gene expression changes (decreased prostate COX-2 mRNA and increased p21 mRNA) with a significant correlation between COX-2 suppression and p21 stimulation and the level of serum isoflavone. These results, supported by in vitro studies, highlight the role of soy isoflavones in PCa chemo-prevention or treatment through modulation of COX-2 and prostaglandin pathway [64]. Moreover, the role of isoflavones in reducing PCa risk was demonstrated by decreased or unchanged PSA and free testosterone in early stage PCa patients in the isoflavone group when compared with placebo [65].

### Broccoli isothiocyanates

Broccoli is a rich source of biologically active isothiocyanates, including sulforaphane and iberin. Importantly, broccoli consumption interacts with glutathione S-transferase mu 1 (GSTM1) genotype modulating signalling pathways associated with inflammation and carcinogenesis in the prostate; the authors also observed changes in TGFβ receptor pathway, insulin signalling, and EGF receptor signalling in men on the broccoli diet. These results provide a mechanistic basis for the effects of broccoli in decreasing PCa risk [66]. Furthermore, a recent study (2020) evaluating chemo-preventive effects of broccoli sprout extract (BSE) demonstrated 40 differentially expressed genes correlating with BSE treatment, including *AMACR* and *ARLNC1*, two genes implicated in PCa development. However, the authors observed no effects on other evaluated markers, such as HDAC activity [67].

### Milk thistle (silibinin)

Milk thistle (*Silybum marianum*) is a therapeutic herb with a 2000 history of use. Milk thistle contains a mixture of flavonolignans known as silymarin while silibinin (also known as silybin) represents its main component [68]. A flavonoid silibinin exerts anti-cancer efficacy including potent inhibitory effects on apoptosis, proliferation, angiogenesis or metastasis associated with prostate carcinogenesis [69]. Recent study also demonstrated the effects of silibinin in decreasing aggressive phenotype in an in vitro model of obesity and PCa. Indeed, silibinin mitigated increased cell growth and invasive capacity of PCa cells exposed to sera of the obese and overweight males. These results indicate the beneficial PCa-protective effects of silibinin in obese or overweight males [70]. Therefore, based on the potent results of preclinical anti-cancer evaluations, silibinin advanced into clinical trials [71]. The evidence of initial clinical evaluations of the effects of silibinin in advanced PCa patients demonstrated oral silybin-phytosome, a commercially available formulation that contains silibinin, in a dose of 13 g/daily delivered in three divided doses to be safe and well tolerated [72]. Moreover, a phase II study revealed that the same dose oral silybin-phytosome achieved high blood concentrations transiently but low levels in prostate tissue of patients with localised PCa [73].

Table 1 provides a detailed overview of the above-discussed effects of phytosubstances/plant-based interventions in primary PCa care as well as the summary of potential adverse events associated with the intervention and major study limitations that need to be carefully evaluated when interpreting the results and proposing a possible implementation into clinical practice.

**Table 1:** Phytochemicals or plant-based interventions in PCa primary care

Phytosubstance/plant-based supplement (dosage)	Study design	Year	Study participants (n = number)	Effects/results	Adverse events of phytosubstance	Major study limitations	Ref
Plant foods (green vegetable, cruciferous vegetable, tomatoes, beans) and whole-grain breads	Data from three case–control studies	1999	Incident PCa cases (n = 617) and controls (n = 636)	Reduced PCa risk	Not available	Multiple comparisons — some findings could occur as significant by chance	[28]
Fruit (cryptoxanthin)				Enhanced PCa risk, independent of antioxidant vitamin intake			
Legumes (not limited to soy) and certain vegetables	Multicentre, multiethnic, case–control study	2000	Confirmed PCa cases (n = 1619) and controls (n = 1618)	Legumes and certain categories of vegetables may protect against PCa	Not available	Not available	[29]
Fruit, vegetable, vitamin A	Follow-up on cohorts of former workers and residents of Wittenoom Gorge since 1975, to document the epidemiology of asbestos-related diseases	2008	PCa cases (n = 1985)	Decreased PCa risk with increasing intake of vegetable rich in vitamin C (bell peppers and broccoli); fruit, other vegetable, vitamin A not observed a strong factor in PCa development	Not available	Analysed ‘total fruit and vegetable’ intakes analysed may not be directly comparable to typical definitions of total fruit and vegetable intakes; Repeated assessments of dietary intake would improve the study; Required careful interpretation (some results may arise by chance)	[30]
Green tea	Case–control study (epidemiological study)	2004	Adenocarcinoma of prostate cases (n = 130) and controls (n = 274)	Declined PCa risk with increasing frequency, duration, and quantity of green tea vs controls	Not available	Not available	[33]
Polyphenon E (800 mg of EGCG and lesser amounts of other GTC, totally 1.3 g of tea polyphenols/day) administered during the interval between prostate biopsy and radical prostatectomy	Open-label, single-arm two-stage phase II clinical trial	2009	Men with positive prostate biopsies (n = 26)	Decreased PSA, VEGF, HGF with no elevation in liver enzymes	No adverse effects (only 1 patient reported mild nausea)	Not available	[34]
Green tea (6 cups/day) or water (control) prior to radical prostatectomy	Randomised exploratory, open label, phase II trial	2015	Men diagnosed with PCa (n = 113) prior to radical prostatectomy randomised into brewed green tea, black tea, or water control	Decreased NFκB in radical prostatectomy tissues, reduced urinary 8OHdG, decrease in serum PSA vs control	No serious adverse event reported	Not blinded study	[37]
Polyphenon E (800 mg of EGCG/day) or placebo for 3–6 weeks until the day before surgery	Randomised, double-blind, placebo-controlled trial	2012	Men with PCa scheduled to undergo radical prostatectomy (n = 50)	Low bioavailability and/or bioaccumulation of green tea polyphenols in prostate tissue; Insignificant changes in PSA, serum insulin-like growth factor, oxidative DNA damage in blood leukocytes	Well tolerated, minimal adverse events (nausea, diarrhoea, headache, 1 patient had a mild ALT elevation)	Short duration of intervention	[38]
Polyphenon E (400 mg of EGCG/day) for 1 year	Placebo-controlled, randomised clinical trial	2015	Men with HGPIN and/or ASAP (n = 97)	EGCG accumulated in plasma; no effects on PCa prevention	Well tolerated	Low completion rate	[39]
Quercetin and green tea (1 g of green tea extract with 800 mg of quercetin or placebo (green tea + placebo) for 4 weeks	Prospective randomised, open label, parallel two arm intervention study	2020	Men scheduled for prostatectomy (n = 31)	No significant increase in EGCG or EGC or decrease in GTP methylation in prostate tissues	No serious adverse effects	Not reported participant food intake (foods containing quercetin or green tea)	[40]
EGCG (600 mg/day) and/or fish oil or placebo	Double-blinded, randomised controlled trial	2016	Men scheduled for repeat prostate biopsy following an initial negative prostate biopsy (n = 89)	No significant changes in FAS or Ki67	No grade ≥ 3 adverse events	Limited sample size, hospital-based design among men scheduled for repeat prostate biopsy may restrict the generalizability of results	[41]
Lycopene and green tea (6 months)	ProDiet randomised controlled trial	2019	Men with raised PSA levels but PC-free (n = 128)	Lycopene lowered pyruvate levels → suggested effects on reduced PCa risk	Not available	ProDiet RCT originally designed to test the feasibility of a dietary intervention (not to detect an effect of the intervention on metabolite levels), small sample size,	[44]
Lycopene (15 mg/day) or placebo for 6 months	Randomised clinical pilot study	2008	Patients with benign prostate hyperplasia (n = 40)	Inhibited disease progression vs placebo	Well tolerated, no adverse events	Not available	[45]
Profluss® (Serenoa repens + selenium + lycopene)		2013	Patients with benign prostate hyperplasia and/or PIN/ASAP (n = 168)	Anti-inflammatory effects	Not available	Lack of placebo controlling	[46]
Red/yellow tomato paste, purified lycopene (yellow and red tomato paste 200 g/d, which provided separated by 2 weeks of washout, in a parallel design first group purified lycopene 16 mg/d for 1 week and second group placebo)	Randomised, single-blinded crossover study for tomato paste studies and a parallel study for lycopene studies, ex vivo study (incubation of LNCaP cells with sera from healthy volunteers)	2010	Healthy men (n = 30)	Upregulated IGFBP-3 and Bax/Bcl-2 ratio and decreased cyclin-D1, p53, and Nrf-2 after cell incubation with sera from health men who consumed red tomato paste	No side effects reported	Not available	[47]
Tomato consumption (canned and cooked) more than 4 times/week	Prospective study (food frequency questionnaire)	2020	Incident cases of PCa (n = 1 226)	Canned and cooked tomatoes may reduce PCa risk (more available lycopene)	Not available	Dietary habits information only from the enrolment questionnaire (repeated measures not provided), not distinguishing between molecular PCa subtypes, relatively low number of aggressive PCa limits power to evaluate risk with good precision	[48]
Tomato sauce	Prospective cohort of men from the Health Professionals Follow-Up Study (food frequency questionnaire)	2016	n = 46 719	Tomato sauce may play a role in *TMPRSS2:ERG*-positive PCa reduction	Not available	Possible misclassification of diet, restricting cases to men treated with radical prostatectomy rendered the entire population of men who did not develop PCa an inappropriate comparison group	[49]
Tomato-enrich diet, lycopene (15 mg capsules/day), or green tea (600 mg/day) for 6 months	Pilot, randomised-controlled trial	2019	Men with PSA between 2.0 and 2.95 ng/ml or negative biopsies (n = 266)	No effects on serum levels of IGF-I, IGF-II, IGFBP-3, or IGFBP-2	Not available	Trial not set up to investigate IGFs as a primary outcome and designed as a feasibility pilot study	[50]
Lycopene (35 mg/day), green tea catechins (600 mg/day), and selenium (55 µg/day) or placebo for 6 months	Double-blind Phase I–II randomised controlled trial	2015	Men with primary multifocal HGPIN and/or ASAP (n = 60)	Higher incidence of PCa at re-biopsy and microRNAs associated with PCa progression vs placebo	Well tolerated	Small number of patients, simultaneous use of three compounds (not allowed precise evaluation of each substance, absence of PCa family history), not performed molecular analysis	[51]
Selenium and lycopene or control for 1 year	Post-hoc analysis of the Procomb trial	2017	Patients who underwent prostate biopsy when ≥ 4 ng/ml and/or PCa suspicion (n = 209)	No detrimental effects on increasing PCa risk; no protective effects	Not available	Lack of measurement of serum levels of selenium or other micronutrients, low rate PCa diagnosed	[52]
Serum lycopene	Nested case–control study in the Prostate Cancer Prevention Trial, a placebo-controlled trial	2011	PCa cases (n = 1683) and controls (n = 1751)	No evidence on association between serum lycopene and PCa	Not available	Not available	[53]
Lycopene-rich tomato extract (30 mg/day) for 6 months	Phase II randomised, double-blind, placebo-controlled trial	2015	Men with HGPIN (n = 58)	Large differences in serum lycopene but no treatment effects	Not available	Small size and restricted statistical power, presence of HGPIN as an endpoint	[54]
Lycopene-rich tomato supplement (30 mg of lycopene/day)	Phase II trial	2007	Androgen-independent PCa patients (n = 46)	Not effective in androgen-independent PCa	Less severe — appeared more plausibly related to lycopene (diarrhoea, nausea, abdominal distension, flatulence, vomiting, anorexia, dyspepsia)	Stable PSA in several patients (unclear whether due to lycopene), PSA decline as primary endpoint	[55]
Retinol and α-carotene (serum)	Nested case–control study – data from PCPT, a multicentre, randomised, placebo-controlled SWOG-coordinated trial	2015	n = 18,880	Increased PCa risk in men with higher level of serum retinol and α-carotene	Not available	Small number of high grade cancers, limited differences by race or ethnicity	[56]
β-carotene (30 mg/day) and retinyl palmitate (25,000 IU/day) for lung cancer prevention	Randomised controlled trial	2009	CARET participants	Increased PCa risk associated with high-dose β-carotene and retinyl palmitate plus at least one other dietary supplement	Not available	Only evidence whether participants used or not supplements — inability to investigate which particular place person at a risk, underpowered to examine PCa deaths	[57]
Common circulating carotenoids and retinol	Men from the Prostate Cancer Prevention Trial placebo arm	2022	Men with negative end-of-study biopsy (n = 235)	Not useful in PCa prevention through the modulation of intraprostatic inflammation	Not available	Inability to assess whether circulating carotenoids reflect prostate tissue levels (circulating levels and tissue inflammation not measured concurrently)	[58]
Genistein (30 mg/day) or placebo for 3–6 weeks	Phase 2 placebo-controlled, randomised, double-blind clinical trial	2012	Early PCa patients before prostatectomy (n = 47)	Reduced KLK4 in tumour cells and a non-significant decrease in androgen and cell cycle-related biomarkers vs placebo	Not available	Small number of cases, short time of intervention	[62]
Soy isoflavones (27.2 mg isoflavone aglycones per tablet, 3 tablets/day) or placebo for 2 weeks	Pilot randomised double blind clinical study	2009	PCa patients (n = 25)	Decreased prostate COX-2 mRNA and increased p21 mRNA	Not available	Not available	[64]
Soy isoflavones (soy beverage protein containing 60 mg of genistein) or placebo for 12 weeks	Prospective randomised, placebo-controlled clinical trial	2004	PCa patients (n = 76)	Decreased or unchanged PSA and free testosterone vs placebo	Not available	Not available	[65]
Broccoli (400 g/week) or peas (400 g/week) for 6 months	Parallel, dietary intervention study	2008	Male volunteers with previous diagnosis of HGPIN (n = 22)	Interaction with GSTM1 genotype modulating signalling pathways associated with inflammation and carcinogenesis, changes in TGFβ, insulin signalling, and EGF (decreasing PCa risk)	Not available	Men within both arms exerted significant changes in androgen receptor pathway (but this can be associated with aging independently of diet), informative stratification of global gene expression profiles, other dietary phytochemicals could interact with plasma signalling peptides	[66]
BSE (200 µmol/day) or a placebo for 4–8 weeks	Double-blind, randomised controlled trial	2020	Men scheduled for prostate biopsy (n = 98)	Differentially expressed genes correlating with BSE treatment — *AMACR* and *ARLNC1* (implicated in PCa development)	Bloating, headache, no grade ≥ 3 adverse events	Short treatment duration	[67]
Milk thistle — silybin-phytosome (2.5–20 g/daily in 3 divided doses)	Phase I trial	2007	PCa patients (n = 13)	13 g of oral silybin-phytosome is well tolerated and recommended to phase II dose	Hyperbilirubinemia (grade 1—2 bilirubin elevations in 9 of 13 patients), ALT elevation (grade 3 toxicity) in one patient; no grade 4 toxicity	Not available	[72]
Milk thistle – silybin-phytosome (13 g/daily in 3 divided doses)	Clinical trial	2010	PCa patients planning for prostatectomy (n = 12)	High-dose oral silybin-phytosome achieves high blood concentrations transiently, but low levels in prostate tissue	Mild (diarrhoea, hyperbilirubinemia). One patient developed grade 4 post-operative thrombo-embolic event	Short duration	[73]

*Abbreviations: ALT*, alanine aminotransferase; *ASAP*, atypical small acinar proliferation; *BSE*, broccoli sprout extract; *CARET*, The Carotene and Retinol Efficacy Trial; *COX-2*, cyclooxygenase-2; *DNA*, deoxyribonucleic acid;* EGC*, epigallocatechin; *EGCG*, epigallocatechin-3-gallate; *EGF*, epidermal growth factor; *FAS*, fatty acid synthase; *GSTM*, glutathione S-transferase mu 1; *GTC*, green tea catechins; *GTP*, green tea polyphenols; *HGF*, hepatocyte growth factor; *HGPIN*, high-grade prostatic intraepithelial neoplasia; *IGF*, insulin-like growth factor; *IGFBP*, insulin-like growth factor binding protein; *KLK4*, kallikrein-related peptide 4; *mRNA*, messenger RNA; *NF-κB*, nuclear factor-κB; *PCa*, prostate cancer; *PIN*, prostatic intraepithelial neoplasia; *PSA*, prostate-specific antigen;* TGFβ*, transforming growth factor beta; *VEGF*, vascular endothelial growth factor; *8-OHdG*, 8-Hydroxy-2'-deoxyguanosine; *g*, gram; *mg*, milligram; *μg*, microgram

Based on the above results, despite the original assumptions about the effectiveness of phytochemicals in PCa prevention, we observe very inconsistent results in the accumulation of phytochemicals or their metabolites in prostate tissue and effects on PCa prevention. However, primary care is at the forefront of a paradigm change from reactive to the cost-effective predictive approach in PCa management. The crucial importance of a personalised approach in PCa management can be illustrated with an example of soy isoflavones in PCa risk assessment provided in a study by Ahn-Jarvis et al. (2015) who described that a characterisation of isoflavonoid metabolic phenotypes is essential to decipher heterogeneity in biological responses among individuals in clinical studies. Therefore, such approaches provide a framework to study isoflavone (phytochemical)-metabolizing phenotypes as a strategy for identification of individuals that might benefit or show resistance to cancer preventive strategies using soy (dietary intervention) [74].

## Phytochemicals in PCa management: secondary and tertiary care

Numerous clinical trials evaluate the potential effects of phytochemicals in already diagnosed PCa patients or patients with recurrent or metastatic disease aimed at the evaluation of their impact on the disease progression [35, 75–79]. Indeed, the effective PCa secondary care highlights the need for the differentiation between non-metastatic and aggressive metastatic disease that requires personalised treatment algorithms. Tertiary PCa care mainly focuses on the palliative care [3]. However, current clinical evidence on the effects of phytochemical in palliative PCa care is lacking. The search on medical database provided only the evidence of phytochemicals affecting the adverse events associated with conventional PCa treatment modalities [22–26] — therefore potentially improving the quality of life during therapy.

### A. The anti-cancer effects of phytochemicals on PCa progression

The potential effects of plant-based interventions or phytochemicals in PCa patients have been clinically evaluated for several decades. At the beginning of the twenty-first century, the small study by Saxe et al. (2001) provided evidence on plant-based diet within Mindfulness-Based Stress Reduction (MBSR) intervention decreasing PSA increase and potential in slowing the progression in patients with recurrent PCa [80]. Similar data were concluded in 2006 supporting the role of plant-based diet and stress reduction in attenuation of PCa progression [81]. As provided below, available data provide evidence of clinical trials evaluating the potential effects of other phytosubstances.

#### Carotenoids

Tomato products and lycopene revealed controversial effects in PCa prevention; however, studies evaluating their efficacy in PCa treatment or prevention of the disease progression demonstrated more concise effects. Earlier published study (2001) described interesting results indicating a role of tomato sauce constituents (especially lycopene) in the short-term treatment of PCa demonstrated by reduced leukocyte and prostate tissue oxidative DNA damage and decreased PSA levels in men with the high-lycopene tomato sauce intervention compared to randomly selected patients [35]. Also, Ansari and Gupta (2003) compared the effects of lycopene plus orchidectomy with orchidectomy alone in metastatic PCa. Indeed, orchidectomy plus lycopene resulted in more reliable and consistent reduction in serum PSA, shrinkage of primary tumour and diminution of secondary tumours, improving survival and better relief from bone pain and symptoms of lower urinary tract when compared with orchidectomy alone [75]. Furthermore, dietary intervention with tomato-products alone or combined with selenium and 3-fatty acids for 3 weeks lowered PSA in non-metastatic PCa patients. The authors suggested that the effects may depend on the aggressiveness of the disease and blood levels of lycopene, omega-3 fatty acids, and selenium. Thus, the control of blood concentrations after dietary interventions seems to be important due to the detection of largest PSA reduction in patients with highest lycopene, selenium, and C20:5 n-3 (eicosapentaenoic acid) increase [76]. Moreover, lycopene and soy isoflavones demonstrated activity in PCa patients with PSA relapse disease demonstrated by PSA stabilisation with the conclusion supporting the potential delay in progression of both hormone-refractory and hormone-sensitive PCa. Besides, the authors suggested no additive effects of the two compounds [82]. On the contrary, lycopene exerted no clinical benefits in PCa patients in advanced stages but two-thirds of patients experienced improved or unchanged situation independently of clinical course or PSA [83].

In addition to tomato products, increased plasma level of β–cryptoxanthin, trans-β–carotene, cis-lutein/zeaxanthin, all-trans-lycopene, and α–tocopherol resulted in lower PSA levels in men with biochemically defined PCa recurrence. Also, higher antioxidant score was observed to be related with lower PSA levels. The results highlight the role of phytochemicals in slowing the PCa progression demonstrated through PSA as a marker of disease progression in men with recurrent PCa [84].

#### Flavonoids of soy and red clover

Isoflavone supplementation revealed potential benefits in men with biochemically recurrent PCa after radiation therapy or radical prostatectomy demonstrated through a decline in PSA slope [85]. Moreover, genistein exerted effects on genome-wide DNA methylation and gene expression, specifically differentially methylated sites and expressed genes involved in developmental processes, stem cell markers, proliferation, and transcriptional regulation (*NOTCH3*, *JAG1*, *ADCY4,* and *NEU1*) as well as reduced MYC activity and increased PTEN activity in genistein group; thus affecting molecular pathways of prostate tumorigenesis [86]. Furthermore, genistein in a dose that can be obtained from a diet rich in soy reduced serum PSA level in patients with localised PCa when compared with placebo [77]. Also, soy-based dietary supplementation (soy, isoflavones, lycopene, silymarin, antioxidants) delayed the progression of PSA when compared with placebo in men with PCa history and rising PSA after radical prostatectomy or radiotherapy [87]. Similarly, soy beverage intervention (containing 50–100 mg of isoflavones daily) for 6 months was associated with a declining trend or more than two times prolongation of PSA doubling time in 41% of patients with rising PSA after radical radiation [88]. On the contrary, short-term intervention with soy isoflavone resulted in no significant changes in selected parameters (PSA, testosterone, cholesterol) in patients with localised PCa [78]. Moreover, high-dose aglycone-rich soy extract elevated serum genistein and daidzein levels but no PSA level changes in men with low-volume PCa [89].

Jared et al. (2002) described that dietary red clover-derived isoflavones might be effective in halting PCa progression by inducing apoptosis in low to moderate-grade tumours and also in potential contribution to lowering the incidence in Asian men [90]. In addition to the above-mentioned entrance of milk thistle phytochemicals into PCa research [72, 73], another study revealed potent efficacy of silymarin (silibinin) against PCa progression. Silymarin combined with selenium administered in patients after radical prostatectomy, reduced low-density lipoprotein and total cholesterol, two markers related to PCa progression [91].

#### Pomegranate polyphenols

Pomegranate is a rich source of polyphenolic compounds, including tannins, anthocyanins, and flavonoids [92]. Pomegranate extract exerted an effect on ≥ 6-month increases in PSA doubling time (PSADT) without adverse effects in men with primary PCa. Indeed, almost one-half of patients who underwent primary therapy for localised PCa is associated with rising PSA levels, indicating PCa recurrence. Gleason scores, time from local treatment to biochemical recurrence, and PSADT functions as a predictor of metastasis-free survival and overall survival. PSADT can be suggested as a predictive factor of PCa progression [93]. However, PSADT is still a controversial primary endpoint in clinical trials [79]. On the contrary, the administration of pomegranate extract before radical prostatectomy revealed no significant changes in the oxidative stress biomarker — 8-OHdG. However, the hypothesis of the protective effect of pomegranate extract against oxidative damage was supported by the capability of its metabolite — Urolithin A of absorption and accumulation in prostate tissues, while high Urolithin A levels correlated with lower 8OHdG levels [94]. Nevertheless, the results from a recent study by Jarrard et al. (2021) concluded that pomegranate compounds could affect biomarkers of oxidative stress demonstrated by reduced 8OHdG and androgen receptor expression in prostate tumour associated with pomegranate fruit extract in men with organ-confined, favourable-risk PCa [95]. In comparison with pomegranate impact discussed above in men with rising PSA following initial PCa therapy [93], daily pomegranate demonstrated no effect on PSA levels in recurrent and advanced PCa patients compared with placebo [96]. In addition, MuscadinePlus, a preparation of pulverised muscadine grape skin, did not prolong PSADT in biochemically recurrent PCa patients [79].

The assumption of potent anticancer effectiveness of individual polyphenol-rich foods was extended by Thomas et al. (2014) who evaluated the effects of an oral capsule with a combination of pomegranate, green tea, broccoli, or turmeric in men with localised PCa either with primary active surveillance (AS) or with watchful waiting (WW) after previous interventions. Finally, the results revealed the beneficial but short-term effect of the capsule containing pomegranate, green tea, broccoli, and turmeric on PSA in men with AS or WW [97].

#### Broccoli phytochemicals — sulforaphane

Glucoraphanin-rich broccoli soup consumption for a year affected gene expression in men on active surveillance, while these changes were consistent with a reduced risk of PCa progression [98]. Also, sulforaphane demonstrated promising results in decreasing PSA progression in PCa patients and biochemical recurrence after definite radical prostatectomy [99]. However, Alumkal et al. (2015) described sulforaphane-rich extracts not being associated with ≥ 50% PSA decline in most recurrent PCa patients conducted in the study. Nevertheless, the authors recommend performing studies evaluating higher doses of the substance [100].

#### Green tea

Patients with androgen-independent prostate carcinoma are associated with limits in treatment options and limited life expectancy. Therefore, it is essential to introduce novel treatment strategies for these patients. However, evaluated green tea exerted limited anticancer capacity demonstrated by a decline in PSA among patients with androgen-independent prostate carcinoma that were asymptomatic and manifested progressive PSA elevation with hormone therapy [101].

#### Flaxseed and curcumin

Flaxseed is a rich source of the plant lignans secoisolariciresinol and matairesinol that are, after ingestion, converted by aerobic intestinal microflora into the enterolignans, enterolactone, and enterodiol. These are suggested to possess potent anticancer effects. Indeed, flaxseed-derived enterolactone is inversely associated with the proliferation of tumour cells in men with localised PCa and possible reduction in angiogenesis [102].

In addition, oral curcumin intake suppressed PSA elevation but had no effects on PCa patients’ overall off-treatment duration of intermittent androgen deprivation (IAD) [103].

Table 2 shows a detailed overview of the above-discussed results of clinical trials evaluating the potential effectiveness of phytosubstances/plant-based interventions in secondary PCa care — especially in individuals with localised, recurrent, or advanced PCa. The Table also includes the data about the study limitations or adverse events associated with the interventions, information that is essential to provide a complex overview of the significance of clinical trials for the clinical practice or future research.

**Table 2:** Clinical evaluations of phytosubstances/plant–based interventions on PCa secondary care

Phytosubstance/plant-based supplement (dosage)	Study design	Year	Study participants (n = number)	Effects/results	Adverse events of phytosubstance	Major study limitations	Ref
Plant-based diet within MBSR intervention for 4 months	Non-randomised clinical trial	2001	PC patients with biochemical recurrence after prostatectomy (n = 10)	↓ rate of PSA increase	Any adverse events described	Small sample size, lack of randomisation, a short period of intervention	[80]
Plant-based diet and stress reduction for 6 months		2006	Recurrent PCa patients (n = 14)	↓ rate of PSA increase	Not available	Small sample size, a short period of intervention, lack of randomised control group	[81]
High-lycopene tomato sauce-based pasta dishes (30 mg of lycopene/day) for 3 weeks	Non-randomised, whole-food intervention arm of an ongoing placebo-controlled clinical trial for the evaluation of lycopene as an in vivo antioxidant	2001	PC patients preceding radical prostatectomy (n = 32)	↓ leukocyte and prostate tissue oxidative DNA damage, ↓ PSA levels vs randomly selected patients	Minor gastrointestinal problems (3 of 32 patients)	Small sample size, more robust analysis required	[35]
Lycopene plus orchidectomy (starting at the day of orchidectomy, 2 mg/twice a day) for 6 months	Clinical trial	2003	Patients with metastatic PCa (n = 54)	More reliable and consistent reduction in serum PSA, shrinkage of the primary tumour and diminution of secondary tumours, improved survival and better relief from bone pain and symptoms of lower urinary tract vs orchidectomy alone	No adverse effects	Appropriate long-term randomised studies are required	[75]
Lycopene-rich tomato intervention (tomato products containing 30 mg lycopene per day, or tomato products plus selenium, omega-3 fatty acids, soy isoflavones, grape/pomegranate juice, and green/black tea, or control diet) for 3 weeks	3-arm randomised controlled trial	2017	Non-metastatic PCa patients (n = 79) prior to curative treatment	Lowered PSA (tomato products alone or in combination with selenium and n-3 fatty acids)	No side effects (only 1 patient discontinued the fish oil supplement intake due to regurgitation)	Products available in Norway — might not reflect the content of lycopene in products of other countries	[76]
Lycopene and soy isoflavones, tomato extract capsule (15 mg of lycopene alone) or together with a capsule (40 mg of soy isoflavone mixture) twice/day max for 6 months	Phase II clinical trial	2007	PC patients with 3 successive rising PSA levels or a minimum PSA of 10 ng/ml at 2 successive evaluations before starting therapy (n = 71)	Stabilisation of PSA levels	Not available	Small sample size, lack of stratification for prognostic factors, lack of a placebo arm	[82]
Lycopene (15 mg/day) for 6 months	Prospective, open phase II pilot study	2009	Patients with progressive hormone refractory PCa (n = 18)	No clinical benefits	Well tolerated	Not available	[83]
Diet and physical activity (plasma carotenoids and tocopherols) for 6 months	Intervention trial	2015	Recurrent PCa patients (n = 39)	Plasma level of α–tocopherol, β–cryptoxanthin, trans-β–carotene, cis-lutein/zeaxanthin, and all-trans-lycopene → lower PSA	Not available	Small sample size, short duration, and the lack of plasma carotenoid and tocopherol data at 6 months (prohibited evaluation of temporal associations)	[84]
Isoflavone supplementation (soy milk containing 47 mg of isoflavonoid per 8 oz serving three times/day) for 12 months	Open-labelled, Phase II, non-randomised trial	2008	Patients with rising PSA after prior local therapy (n = 20)	A decline in slope of PSA	Minimal (1 patient — diarrhoea)	A small study that was terminated early due to poor accrual, no control group, and therapy was neither randomised nor blinded, with serum PSA as the primary endpoint	[85]
Genistein (30 mg/day) or placebo for 3–6 weeks before prostatectomy	Randomised, placebo-controlled, double-blind clinical trial	2017	PCa patients (n = 20)	Differentially methylated sites and expressed genes (between genistein and placebo group) involved in developmental processes, stem cell markers, proliferation, and transcriptional regulation (*NOTCH3, JAG1*, *ADCY4*, and *NEU1*). Reduced MYC activity and increased PTEN activity in genistein group	Not available	Small number of patient samples	[86]
Genistein (dose that can be obtained from a diet rich in soy) or placebo for 3–6 weeks before prostatectomy	Placebo-controlled, block-randomised double-blind phase 2 study	2011	Patients with localised PCa before radical prostatectomy (n = 54)	Decreased serum PSA level, no effects on hormones in genistein group vs placebo	No adverse effects of clinical significance, only mild (in genistein arm 5 events — 3 gastrointestinal, 1 cardiovascular, and 1 general)	A small number of patients	[77]
Soy-based dietary supplement (soy, isoflavones, lycopene, silymarin, antioxidants) for 10 weeks, 4 weeks wash-out period, and 10 weeks	Randomised, double-blind, placebo-controlled crossover study	2005	Patients with PCa history and rising PSA after radical prostatectomy (n = 34) or radiotherapy (n = 15)	Delayed PSA progression vs placebo	Few adverse events that occurred were not related to the use of the dietary supplement	A limited number of patients, more extensive studies, better standardisation, and characterisation of the effects of each compound on PCa biology are required for recommendations for the general public	[87]
Soy beverage (500 ml/day containing app. 50–100 mg of isoflavones) for 6 months	Phase II trial	2010	PC with rising PSA after radical radiation (n = 34)	Declining trend or more than 2 times prolongation of PSA doubling time in 41% of patients	Well tolerated	Not measuring the intake of other phytochemicals that could potentially affect PSA, the use of PSADT as a surrogate endpoint	[88]
Soy isoflavone capsules (82 mg/day of total isoflavones) or placebo for 4 weeks before prostatectomy	Double-blinded, randomised, placebo-controlled trial	2013	Localised PCa patients (n = 86)	No changes in serum total testosterone, free testosterone, total oestrogen, oestradiol, PSA, and total cholesterol	Safe, only mild adverse effects (gastrointestinal and general)	Lack of stratification of results based on Gleason score, pathologic stage, or PSA, a small number of tissue samples analysed	[78]
High-dose aglycone-rich soy extract — treatment group (supplement containing 450 mg genistein, 300 mg daidzein, and other isoflavones/day) for 6 months or placebo	Double-blind, placebo-controlled, randomised trial	2010	Men with low-volume PCa (n = 53)	Elevated serum genistein and daidzein levels; no changes in PSA level	Well tolerated (only loose stools are the most common complaint from a small number of men)	A small number of patients	[89]
Red clover-derived isoflavones (160 mg of isoflavones daily consisting of 4 tablets/day containing 40 mg of standardized red clover-derived isoflavones) for 20 days (median)	Non-randomised, non-blinded trial with historically matched controls from archival tissue	2002	Treated and untreated control PCa specimens (n = 36)	Induced apoptosis (higher apoptosis in radical prostatectomy specimens from treated patients vs control)	No adverse effects reported	Observational study on a small cohort of Australian men	[90]
Silymarin (570 mg) and selenium (240 µg) or placebo for 6 months	Placebo-controlled double-blind clinical trial	2010	PC patients after radical prostatectomy (n = 37)	Reduced low-density lipoprotein and total cholesterol	No adverse effects reported	Not available	[91]
Pomegranate extract (1 or 3 g) for up to 18 months	Randomised, multi-centre, double-blind phase II, dose-exploring trial	2013	Men with rising PSA following initial PCa therapy (n = 104)	Lengthened PSADT independently of dose and without adverse effects	No adverse effects	Lack of placebo arm (placebo-controlled trials needed, e.g. NCT00732043 or NCT00719030)	[93]
Pomegranate extract (2 tablets/day — each capsule contains 1000 mg of pomegranate extract powder that contains up to 600 mg of polyphenol from extract) or placebo	Phase II, randomised, double-blind trial	2013	PC patients prior to radical prostatectomy (n = 70)	No significant changes in 8OHdG levels; however, Urolithin A capable of absorption and accumulation in prostate tissues -high Urolithin A level correlated with lower 8OHdG levels	No serious adverse effects, only grade I (mostly nausea, diarrhoea)	The primary end-point is an intermediate surrogate biomarker end-point (unclear clinical relevance of 8OHdG), the number of men included was modest (limiting statistical power), the duration of pomegranate extract therapy was short and the dose was modest)	[94]
Pomegranate fruit extract (1000 mg capsule/day) or placebo for 52 weeks	Randomised, placebo‐controlled trial	2021	Active surveillance patients—men with organ-confined, favourable-risk PCa (n = 30)	Reduced 8OHdG and androgen receptor	Side effects felt to be unrelated to the administration of the study drug	No preliminary data on the calculation of the sample size for each treatment arm upon which to estimate treatment effect size, period of administration (1 year) is relatively short to generate large effects, 35.7% and 40% of patients in the treatment arms did not display carcinoma at the end of study biopsies	[95]
Pomegranate juice (500 ml/day of juice or placebo for 4 weeks), then all patients pomegranate juice (250 ml/day) for 4 weeks	Phase IIb, double-blinded, randomised placebo-controlled trial	2013	Recurrent and advanced PCa patients (n = 97)	No effect on PSA	Well tolerated (bowel disturbances most frequently reported)	Certain heterogeneity of the included patient cohort	[96]
Polyphenol-rich oral capsule 3 times/day (containing broccoli powder 100 mg, turmeric powder 100 mg, pomegranate whole fruit powder 100 mg, green tea 5:1 extract 20 mg equivalent to 100 mg of green tea) for 6 months	A double-blind, placebo-controlled randomised trial	2014	Men with localised PCa with AS or WW (n = 199)	Short-term favourable effect on PSA rise vs placebo	Gastrointestinal events	No proven long-term effects	[97]
MuscadinePlus (muscadine grape skin extract) for 12 months	12-month, multicentre, placebo-controlled, two-dose, double-blinded trial	2018	Men with biochemically recurrent PCa (n = 125)	No prolongation of PSADT	Adverse effects judged to be unrelated or unlikely related to the study product	Dependence on PSADT as the primary endpoint	[79]
Glucoraphanin-rich broccoli soup (300 ml) consumption for a year	3-arm parallel randomised double-blinded intervention study	2019	Men on active surveillance (n = 61)	Changes in gene expression in men on active surveillance while these changes were consistent with reduced risk of PCa progression	Not available	Small sample size, not met target recruitment to achieve the original power estimation, biopsies analysed were all considered nonneoplastic, based on directly adjacent histology	[98]
Sulforaphane (60 mg) for 6 months followed by 2 months without treatment	Double-blinded, randomised, placebo-controlled multicentre trial	2015	PC with increasing PSA levels after radical prostatectomy (n = 78)	Promising results on the effectiveness in decreasing PSA progression in PCa with biochemical recurrence after definite radical prostatectomy	Gastrointestinal adverse events (bloating)	Use of PSA as an endpoint (but PSA is the only available follow-up marker in this setting)	[99]
Sulforaphane-rich extracts (200 μmoles/day) for max 20 weeks	Single arm trial	2015	Recurrent PCa patients (n = 20)	No significant effects on PSA reduction	Safe with no grade II adverse events, gastrointestinal disorders (bloating, diarrhoea, dyspepsia, flatulence)	Lack of a placebo control arm limits interpretability	[100]
Green tea (6 g/day)	Phase II clinical trial	2003	Asymptomatic PCa patients with manifested progressive PSA elevation with hormone therapy (n = 42)	Limited anticancer efficacy of green tea	Well tolerated for the most part (grade 1 or 2 and included nausea, emesis, insomnia, fatigue, diarrhoea, abdominal pain, and confusion), but there were six episodes of Grade 3 toxicity (insomnia, confusion, diarrhoea, fatigue, and abdominal pain) and one episode of Grade 4 toxicity (confusion)	Not available	[101]
Oral curcumin (1440 mg/day) or placebo for 6 months	Randomised, double-blind, placebo-controlled trial	2019	PC patients who received IAD (n = 80)	No effect on overall off-treatment duration of IAD; suppressed PSA elevation	Adverse events were higher in the placebo group	Included subjects were from different clinical situations (biochemical recurrence after localized treatments and metastatic disease)	[103]
Flaxseed, low-fat diet, or both for ∼30 days before surgery	Data from our previous multisite phase II randomised controlled trial (NCT00049309)	2013	Men with PCa before prostatectomy (n = 161)	Evidence of plant lignans via flaxseed supplementation to inhibit cancer growth (proliferation) — inverse correlation between total urinary enterolignans and enterolactone correlated with Ki67 (significant); possible reduction in angiogenesis (non-significant)	Not available	Not available	[102]

*Abbreviations*: *AS*, active surveillance; *DNA*, deoxyribonucleic acid; *IAD*, intermittent androgen deprivation;* MBSR*, Mindfulness-Based Stress Reduction; *PCa*, prostate cancer; *PSA*, prostate-specific antigen; *PSADT*, PSAdoubling time; *WW*, watchful waiting; *g*, gram; *mg*, milligram; *μg*, microgram; *ml*, millilitre

### B. Stimulating effects of phytochemicals on anti-cancer chemo- and radiotherapy in PCa management

Currently, incurable metastatic PCa is considered a therapeutic challenge. Most advanced PCa patients have a good initial response to androgen deprivation therapy with luteinizing hormone-releasing hormone analogs, orchiectomy, and/or testosterone receptor antagonists. But patients consequently progress into castration-resistant PCa characterised by a median survival of 2–2.5 years. The disease in most patients, however, further progress despite anti-androgenic therapy, and these patients require the administration of cytotoxic agents, such as docetaxel [21].

The capacity of phytochemicals to potentially improve the efficacy of conventional anti-cancer treatment has been described in various cancer types [9, 104]. The study evaluating the combination of docetaxel, prednisone, and curcumin in patients with castration-resistant PCa described good tolerability and patient acceptability [22]. However, a recent study by Passildas-Jahanmohan et al. (2021) observed no effects of adding curcumin to treatment strategies (docetaxel) for patients with castration-resistant PCa in improving patient outcome and prognosis [23]. On the contrary, lycopene plus docetaxel exerted favourable effects in metastatic castrate-resistant PCa patients. The synergistic activity of lycopene with docetaxel is based on the effects on downregulation of IGF-I signalling inhibition and decrease in survivin expression. Indeed, previous evidence from PCa models describes that lycopene could suppress IGF-I, thus promoting docetaxel response [21].

### C. Phytochemicals mitigate adverse effects of chemo- and radiotherapy

Evidence supports the role of phytochemical in mitigating adverse effects of conventional anti-cancer treatment modalities, e.g. radiotherapy or chemotherapy that are usually associated with various adverse events [9]. Indeed, ellagic acid reduced toxicity induced by chemotherapy (neutropenia) in hormone-refractory PCa patients. Moreover, the results also support the potential anti-cancer action of ellagic acid due to the observed decrease in serum PSA and a positive trend toward objective response and overall survival in the experimental group compared to the control [24].

Radiotherapy represents a vital PCa treatment modality [25]. External beam radiation therapy is associated with acute and subacute toxicities, including intestinal and urinary adverse effects and erectile dysfunction. However, Ahmad et al. (2010) concluded that soy isoflavones in conjunction with radiation therapy could reduce urinary, sexual, and intestinal adverse effects of radiation therapy in PCa patients [26]. Besides, up to 75% of patients receiving radiotherapy develop symptoms related to acute radiation-induced proctitis. The evaluation of potential effects of nano curcumin in PCa patients undergoing radiotherapy has not concluded effects neither to prevent and/or mitigate radiation-induced proctitis nor in radiation-induced cystitis, duration of radiation toxicities, hematologic nadirs, and tumour response. These results provide the translational insight to bridge the gap between clinical and laboratory practice despite any significant effect observed. Therefore, the authors conclude that studies with many patients and long-term pre-treatment with nano curcumin could clarify if the curcumin functions as radiosensitizer or radioprotector [25].

Table 3 provides a detailed overview of the above-discussed clinical evaluations of the effects of phytosubstances/plant–based on the anti-cancer effectiveness or mitigating the adverse effects of conventional PCa therapeutic modalities, with the overview of potential adverse effects of the phyto-interventions and significant study limitations that need to be carefully evaluated when interpreting results of the trials and their potential implementation into clinical practice.

**Table 3:** Clinical evaluations on the effects of phytosubstances/plant–based intervention on conventional treatments of PCa

Phytosubstance/plant-based supplement (dosage)	Study design	Year	Study participants (number)	Effects/results	Adverse events of phytosubstance	Major study limitations	Ref
Docetaxel, prednisone, and curcumin (6000 mg/day–12 curcumin capsules/day for 7 consecutive days)	Non-randomised, open-label, phase II trial	2016	Patients with progressing castration-resistant PC	High response rate, good tolerability, and patient acceptability (tumour objective response in 40% and a PSA response in 59% of men)	Well tolerated curcumin, without systemic toxic effects	Single-arm, non-randomised design of the study, the low number of patients	[22]
Docetaxel plus curcumin (6 g/day) or docetaxel plus placebo in first-line treatment for 7 consecutive days every 3 weeks	Double-blind, randomised, phase II study	2021	Patients with metastatic castration-resistant PCa (n = 50)	No effects of adding curcumin to treatment strategies in improving patient outcome and prognosis	Most common: anaemia, asthenia, diarrhoea, and alopecia. Nothing relevant was noted between the two groups of patients, except less lymphopenia and less hypocalcaemia in the experimental arm	Small sample size, titration of curcumin performed for only a few patients	[23]
Docetaxel every 21 days plus lycopene daily (30 mg/day)	Interventional Phase II clinical trial	2021	Metastatic castrate-resistant PCa patients (n = 13)	Favourable effects, synergistic activity of lycopene with docetaxel (downregulation of IGF-I signalling inhibition and decrease in the expression of survivin)	Not available	Small sample size	[21]
Soy isoflavones (200 mg/day) or placebo for 6 months, beginning with the first day of radiation therapy	Double-blind, placebo-controlled, randomised trial	2010	PC patients (n = 42)	Reduced urinary, sexual, and intestinal adverse effects of radiation therapy	Not available	A small number of subjects, study coordinators should assist patients with the administration of study questionnaires for better compliance	[26]
Ellagic acid (180 mg/day) throughout the chemo-therapy cycles and during the period between cycles	Clinical trial	2005	Hormone refractory PCa patients (n = 48) on standard chemo-therapy using vinorelbine and estramustine phosphate	Reduced toxicity induced by chemo-therapy (neutropenia)	Not available	Not available	[24]
Nanocurcumin (120 mg/day) or placebo 3 days before and during radiotherapy	Randomised, double-blind, placebo-controlled phase II trial	2019	PC patients (n = 64)	No effect on preventing and/or mitigating radiation-induced proctitis or in radiation-induced cystitis, duration of radiation toxicities, hematologic nadirs, and tumour response	Well tolerated, no drug-related severe adverse effects	Single-centre design (not representing the entire population), a small number of patients, underpowered trial to accept or reject the study hypothesis	[25]

*Abbreviations*: PCa, prostate cancer; g, gram; mg, milligram.

## Conclusions in the framework of predictive, preventive and personalised medicine (PPPM/3PM)

Utilisation of phytochemicals as potent anti-cancer agents represents the cornerstone in implementing novel, highly effective, well-tolerated, safe, and cost-effective measures in multi-faceted anti-PCa protection and disease management.

### Primary care

Effective PCa management requires a paradigm change from reactive to predictive, preventive, and personalised medicine [1]. PCa is a systemic multi-factorial disease that results from an imbalance between excessively accumulated health risks and insufficient protection [4]. To this end, PCa develops over years or even decades via health-to-disease transition. Sub-optimal health conditions are characterised by a reversible damage to health presenting the opportunity for primary care to implement innovative tools of personalised risk assessment followed by cost-effective personalised anti-PCa protection tailored to the individual risks [106]. Contextually, targeted anti-PCa protection is at the forefront of the paradigm change from reactive to the predictive, preventive and personalised approach in PCa management. Phytochemicals are associated with potent anti-cancer activity targeting each stage of carcinogenesis starting with sub-optimal health conditions. For example, their positive effects are demonstrated for stabilising and restoring mitochondrial health quality, which if compromised is strongly associated with sub-optimal health conditions and strong predisposition to aggressive cancer sub-types [105]. An absolute majority of altogether 30 clinically relevant studies dedicated to phytochemicals in the PCa primary prevention which we have identified in the literature, demonstrated positive effects and potentially reduced risks of the disease development such as listed by references [33, 34, 45, 49]. To this end, we do strongly recommend the stratification of affected individuals in sub-optimal health conditions by phenotyping for targeted PCa-prevention and identification of the most effective plant-based treatment options [3, 49, 105, 107].

### Secondary care

A rapid increase in PCa incidence and lack of adequate patient stratification to differentiate between non-metastatic PCa (no necessity for expensive treatments) and aggressive PCa subtypes requiring personalised treatment algorithms contribute to the enormous socio-economic burden currently caused by sub-optimal PCa management [3]. Consequently, risk assessment, patient stratification, targeted prevention of metastatic disease and treatment algorithms tailored to the person are the main pillars of PPPM strategies which would significantly advance secondary care in overall PCa management with potential to reverse current economic trends [3].

The effects of plant-derived phytochemicals were evaluated for secondary care particularly focused on reducing risks of the disease progression. By evaluating randomly selected clinical trials (29 studies in total), almost 70% were identified as demonstrating positive effects of phytosubstances in reducing risks of PCa progression such as listed by references [35, 76]. Moreover, several studies included in our review highlighted supportive and stimulating effects of conventional anti-cancer therapy as well as an evident mitigation of their adverse effects [21–26].

### Tertiary care

Reactive medical services require biggest budgets, in particular dedicated to the last life year of the PCa patients [3]. Therefore, the intention of PPPM concepts is to treat affected individuals at the initial care levels (primary and secondary prevention). Nevertheless, the motivation of palliative care is to make palliative medicine to the management of chronic disease. For reaching the goal, treatment algorithms should consider comprehensive individualised patient profiles utilising big data analysis and machine learning approach [108]. To this end, application of natural compounds based on flavonoids and their nano-technologic derivatives may significantly contribute to improved individual outcomes and extended life expectation in the tertiary care of PCa. Corresponding therapeutic modalities consider their immune-modulating and drug-sensitising effects as well as excellent capacity to increase sensitivity of cancer cells and to reverse cancer resistance against anti-cancer therapies [109].

**Fig. 2:**
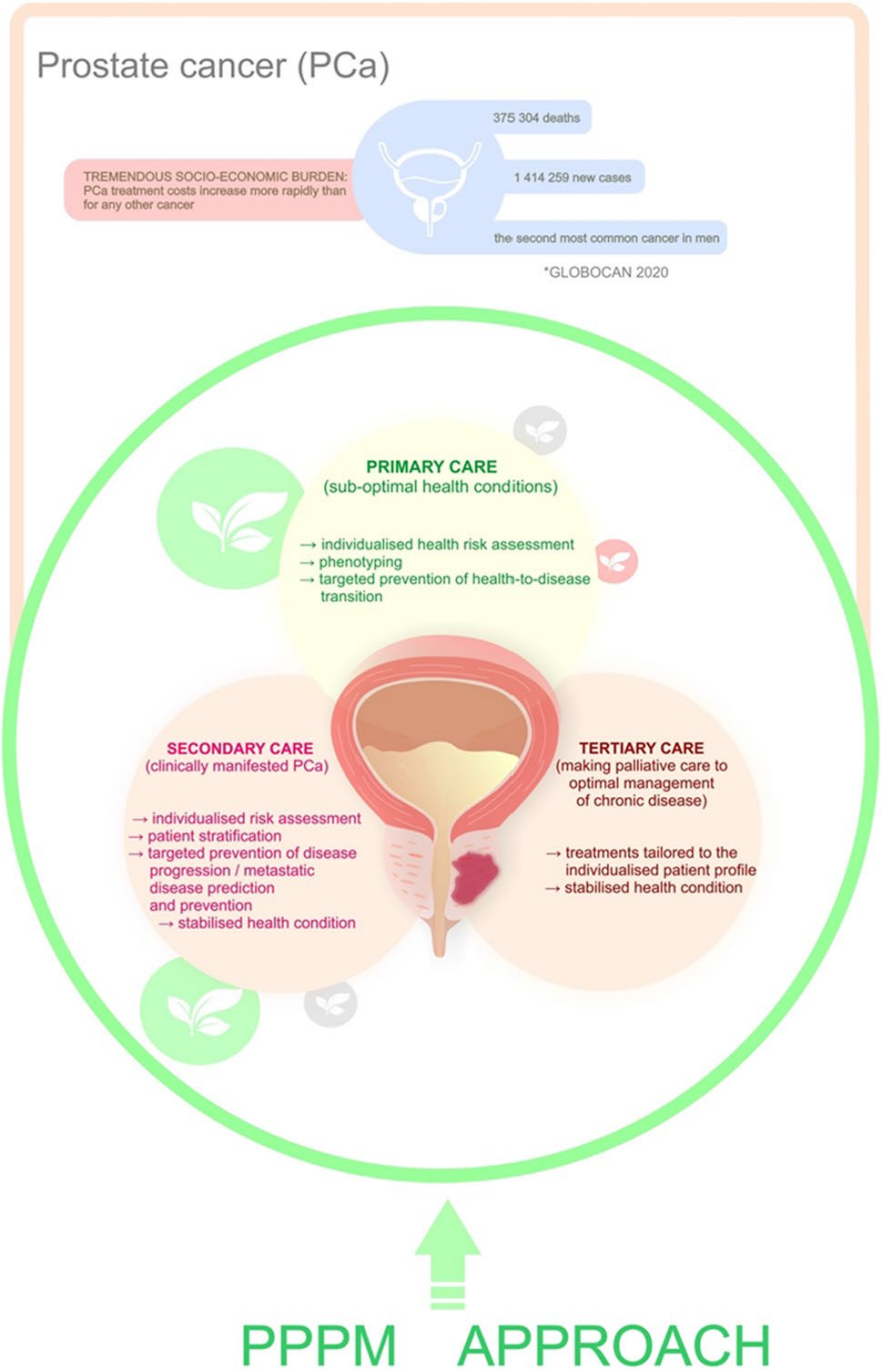
Conclusions in the framework of Predictive, Preventive and Personalised Medicine (PPPM)

Finally, PCa-affected individuals per evidence are highly vulnerable towards COVID-19 infection [3]. Therefore, dual anti-cancer and anti-viral effects of phytochemicals such as these of silibinin are highly relevant for improved PCa management at the level of secondary and tertiary care under pandemic conditions [110]. Silibinin forms a stable complex with SARS-CoV-2 spike protein RBD being capable to interact with the active site of Mpro inhibiting, therefore, viral entry and replication. Further, silibinin may reduce pro-inflammatory effects and endothelial dysfunction by regulating expression patterns of TNF-α, IL-6 and ET-1 in blood plasma [110]. Figure 2 highlights the key concepts of the PPPM approach in primary care (anti-PCa protection) and advanced management of the clinically manifested diseases.

## Data Availability

Not applicable.
